# A Unique Signature for Cancer‐Associated Fibroblasts in Melanoma Metastases

**DOI:** 10.1111/pcmr.70002

**Published:** 2025-02-09

**Authors:** Saskia Tauch, Joschka Hey, Bettina Kast, Nicolas Gengenbacher, Lena Weiß, Melanie Sator‐Schmitt, Sabrina Lohr, Alexander Brobeil, Peter Schirmacher, Jochen Utikal, Hellmut G. Augustin, Christoph Plass, Peter Angel

**Affiliations:** ^1^ Division Signal Transduction and Growth Control German Cancer Research Center (DKFZ‐ZMBH Alliance) Heidelberg Germany; ^2^ Faculty of Biosciences Heidelberg University Heidelberg Germany; ^3^ Division of Cancer Epigenomics German Cancer Research Center (DKFZ) Heidelberg Germany; ^4^ Division of Vascular Oncology and Metastasis German Cancer Research Center (DKFZ‐ZMBH Alliance) Heidelberg Germany; ^5^ European Center for Angioscience (ECAS), Medical Faculty Mannheim Heidelberg University Mannheim Germany; ^6^ DKFZ‐Hector Cancer Institute, University Medical Centre Mannheim Mannheim Germany; ^7^ Institute of Pathology University Hospital Heidelberg Heidelberg Germany; ^8^ Skin Cancer Unit German Cancer Research Center (DKFZ) Heidelberg Germany; ^9^ Department of Dermatology, Venereology and Allergology University Medical Center Mannheim, Ruprecht‐Karl University of Heidelberg Mannheim Germany

**Keywords:** CAF, melanoma, metastasis, SAA, scRNA sequencing

## Abstract

Cancer‐associated fibroblasts (CAFs) represent a central cell population of the tumor microenvironment (TME). Recently, single‐cell RNA‐sequencing (scRNA‐seq) analyses of primary tumors of different cancer entities yielded different classifications of CAF subsets underscoring the heterogeneity of CAFs within the TME. Here, we analyzed the transcriptional signatures of approximately 8400 CAFs and normal fibroblasts by scRNA‐seq and compared genetic profiles of CAFs from murine melanoma primary tumors to CAFs from corresponding melanoma lung metastases. This revealed distinct subsets for primary tumor and metastasis‐specific CAF populations, respectively. Combined with the spatial characterization of metastasis CAFs at the RNA and protein level, scRNA analyses indicate tumor‐dependent crosstalk between neutrophils and CAFs, mediated via SAA3 and IL1b‐related signaling pathways, which can be recapitulated in vitro. Analyzing tissue sections of human patient samples, this interaction was found to be present in human melanoma metastasis. Taken together, our data highlight unique characteristics of metastasis CAFs with potential therapeutic impact for melanoma metastasis.

## Graphical abstract

In this study, we analyzed the transcriptional signatures of CAFs and normal fibroblasts from murine melanoma primary tumors and corresponding melanoma lung metastases. Single‐cell analysis revealed distinct clusters for primary tumor and metastasis CAFs. In lung metastases Saa3 expression is a hallmark of metastasis CAFs. Overall, our data suggest an Il‐1b‐SAA3‐specific crosstalk between CAFs and neutrophils resulting in the formation of protumorigenic NETs. Anakinra may represent a therapeutic option to interfere with this crosstalk.

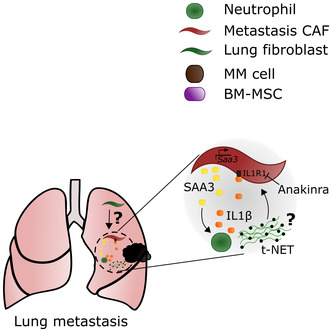

**Summary**

- The tumor microenvironment and cancer‐associated fibroblasts (CAFs), especially, have gained more importance in tumor development and metastasis, during the last decade.
- Here, we show an analysis of melanoma CAFs at the single‐cell level, revealing unique characteristics of metastasis CAFs.
- This data provide not only valuable insights into the role of CAFs in melanoma metastasis but combined with analyses of human patient samples, highlight a potential therapeutic impact of metastasis CAFs for melanoma metastasis.

**Abbreviations**
apCAFs: antigen‐presenting CAFs
AT‐2: alveolar type II cells
BEC: blood endothelial cells
CAFs: cancer‐associated fibroblasts
CitH3: citrullinated histone H3
EMT: mesenchymal transition
EndMT: endothelial‐to‐mesenchymal transition
FACS: fluorescence‐activated cell sorting
iCAF: inflammatory
LEC: lymphatic endothelial cells
MM: malignant melanoma
MPO: myeloperoxidase
myCAF: myofibroblastic
PDAC: pancreatic ductal adenocarcinoma
PDPN: podoplanin
RCC: respiratory ciliated cells
scRNA‐seq: single‐cell RNA sequencing
TME: tumor microenvironment
αSMA: α‐smooth muscle actin

## 1 Introduction

Malignant melanoma (MM) represents a very aggressive form of skin cancer, accounting for over 65% of skin cancer‐related deaths while relating to only about 5% of all skin cancer cases (Davey et al. 2021; Domingues et al. 2018; Ransohoff et al. 2016). This is aggravated by a continuously increasing global incidence (Davey et al. 2021; Schadendorf et al. 2015). Since broadly spread metastasis is the leading cause of MM‐related death, metastasis‐specific characteristics could serve as potential therapeutic targets (Zbytek et al. 2008).

Current models of cancer development and metastasis appreciate the central role of nonmalignant cells from the tumor microenvironment (TME) (Hanahan and Weinberg 2011). Strikingly, cancer‐associated fibroblasts (CAFs) were shown to be implicated in the majority of hallmarks of cancer, and thus, have entered the limelight of cancer research. In the specific context of MM, CAFs support BRAF inhibitor resistance (Hirata et al. 2015) and influence immunotherapy outcome (Wong et al. 2019).

CAFs are thought to resemble activated myofibroblasts, albeit showing a higher degree of heterogeneity (Deyell et al. 2021; Ping et al. 2021). Based on the heterogeneity of CAFs, multiple sources have been described. These include local fibroblasts, mesenchymal stem cells, as well as epithelial and endothelial cells (Quante et al. 2011; Zeisberg et al. 2007; Petersen et al. 2003). Thus, CAFs have been considered to rather be a dynamic cell state than a specific cell type (Madar et al. 2013).

In recent years, multiple publications proposed different classifications of CAFs in a range of tumor entities based on scRNA‐sequencing analysis. Investigating CAFs in MM, immune, desmoplastic, and contractile CAFs were defined in murine B16. F10‐derived primary tumors (Davidson et al. 2020). Albeit, unraveling new insights into CAF populations, most studies focused on CAFs in primary tumors, leaving the critical question of a putative metastasis‐specific targetable CAF signature unanswered.

In this study, we apply scRNA sequencing on primary tumor and lung metastasis samples from an improved mouse model (Gengenbacher et al. 2021) of MM development and metastasis to identify putative metastasis‐specific therapeutic targets for MM.

Noteworthy, we found one metastasis‐specific CAF cluster, which mounts an Il‐1b‐SAA3‐specific connection between immune cells and CAFs. Our data for metastasis‐specific CAFs complement and expand the present scRNA‐sequencing data for CAFs derived from primary tumors and may open the way for metastasis‐specific therapies, targeting CAFs.

## 2 Results

### 2.1 scRNA‐Sequencing Data Mirror the Complexity of the Tumor Stroma

To define metastasis‐specific CAF profiles by scRNA sequencing, we applied the MT‐ret melanoma fragment transplantation model (Gengenbacher et al. 2021) (MT‐ret–derived model) and combined it with mT/mG mice (Gt (ROSA)26Sor^tm4(ACTB‐tdTomato,‐EGFP)Luo/^J) (Muzumdar et al. 2007), generating MM primary tumor samples and corresponding lung metastases, in which engrafted tumor cells are unlabeled while host TME cells constitutively express tdTomato (Figure S1a). To confirm that the vast majority of CAFs are indeed tdTomato^+^, we performed immunofluorescence staining of the fibroblast markers podoplanin (PDPN) and α‐smooth muscle actin (αSMA) on tissue sections of a primary tumor engrafted in a mT/mG mouse (Figure S1b). Indeed, both, PDPN and αSMA, colocalized with tdTomato, verifying that CAFs are effectively labeled and derived from the mT/mG host.

**FIGURE 1:**
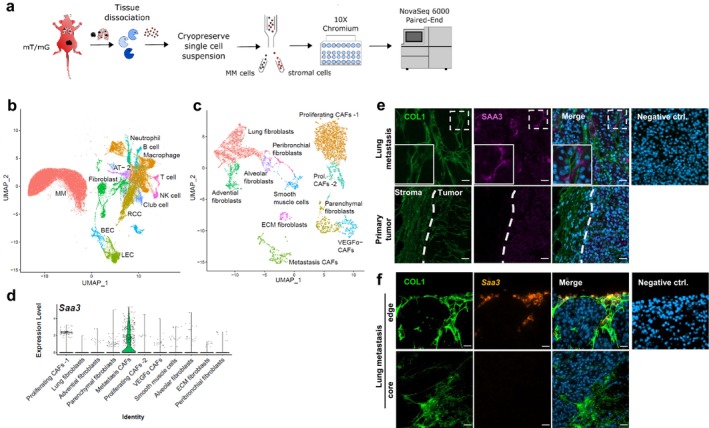
scRNA data mirror the complex cellular composition of primary tumors and metastases. (a) Schematics of workflow. (b) Cell clusters based on 10× Genomics scRNA‐sequencing analysis visualized by uniform manifold approximation and projection (UMAP). Cells are color coded according to cell types. (c) Fibroblast clusters extracted from (b). (d) The violin plot shows the expression of *Saa3* for fibroblast clusters. (e) IF staining of collagen I, and SAA3 on lung metastasis and primary tumor. Time point of metastasis = 6 weeks after primary tumor resection. Dashed line: tumor/TME margin. (f) RNAscopeTM in situ hybridization of *Saa3* and IF staining of collagen I on lung metastasis. Upper line: Leading edge, lower line: Tumor core. Time point of metastasis = 2 months after primary tumor resection. Scale bar = 20 μm.

To circumvent the current lack of a general CAF surface marker and to acknowledge CAF heterogeneity, library preparation was performed with all tdTomato^+^ CD45^−^ cells without further positive selection for fibroblast markers (Figure 1a). Corresponding tdTomato^−^ MM cells were processed in parallel as separate samples. Additionally, cells from normal lung tissues were included as unchallenged control. After quality control, approximately 227,146 cells remained in the dataset, clustering into 12 cell‐type clusters (Figure 1b). Of these, 37,368 cells derived from the unchallenged lung controls, 13,850 cells from the lung metastasis TME, and 9163 cells from the primary tumor TME (Figure S2a). According to commonly known linage markers, we were able to identify MM cells (*Cited1, Cd63, Dct*), fibroblasts (*Mgp, Dcn, Col1a1*), blood‐(BEC; *Pecam, Cd34, Vwf*), and lymphatic endothelial cells (LEC; *Lyve1, Tmem100, Cldn5*), as well as immune cells including macrophages (*Lyz2, Chil3, Ccl6*), neutrophils (*S100a9, S100a8, Il1b*), B cells (*Cd79a, Cd79b, Igkc*), NK cells (*Nkg7, Gzma, Ccl5*), and T cells (*Cd3g, Il7r, Trac*). Lung‐specific cell types like alveolar Type II cells (AT‐2; *Sftpb, Sftpd, Slc34a2*), club cells (*Scgb3a2, Scgb1a1, Scgb3a1*), and respiratory ciliated cells (RCC; *Tmem212, Sec14l3, Ccdc153*) were also present (Figure S2b, Table S1). Collectively, these clusters mirrored the heterogeneity of tumor, metastasis, and lung tissues.

### 2.2 Single‐Cell Analysis Reveals Distinct Clusters for Primary Tumor and Metastasis CAFs

For in‐depth analysis of CAFs, we isolated CAFs and normal fibroblasts in silico based on a larger set of fibroblast marker genes (Figure S3a). This resulted in 11 fibroblast subclusters, containing approximately 8400 cells (Figure 1c). Using previously described marker genes for known fibroblast subtypes, clusters could be assigned to alveolar (*Npnt, Mfap4, Fmo2, Inmt*) and peribronchial (*Hhip, Mustn1, Enpp2*) lung fibroblasts, smooth muscle cells (SMC; *Rgs5, Myh11, Myl9*), and advential (*Pi16* and *Ly6a*) and parenchymal (*Col15a1* and *Fbn2*) fibroblasts. Extracellular matrix fibroblast and VEGFA CAFs were termed according to the upregulation of marker genes related to extracellular matrix (*Tnxb, Col3a1, Col1a2, Fbn1*) and angiogenesis (*Vegfa, Hspa1a, Hspa1b*; Figure S3b, Table S2), respectively. Performing cell cycle analysis based on the ratio of expression levels of genes related to G1, S, or G2/M revealed two highly proliferative clusters, which we labeled proliferating CAFs‐1 and ‐2 (Figure 1c and Figure S3c).

To identify the CAF clusters specifically derived from lung metastases, cells were color coded according to their sample of origin (Figure S3d). This revealed the presence of tissue‐specific normal fibroblast and CAF clusters, but also clusters, which were common for multiple tissue sources. Strikingly, fibroblasts from metastasis formed one defined cluster, which mainly contained metastasis‐derived cells. Thus, this cluster was termed the metastasis CAF cluster. Furthermore, the clusters of alveolar and peribronchial fibroblasts, contained, besides cells from unchallenged lung samples, also cells from metastasis samples (Figure S3d). This is due to the fact that the metastasis samples included a small amount of nontumorigenic adjacent tissue.

### 2.3 *Saa3* Expression is a Hallmark of Metastasis CAFs

To understand the role of metastasis CAFs, we investigated the genes of the metastasis CAF cluster. The signature of metastasis CAFs (*Clu*, *Cxadr*, *Lgals7*, *Calca*, *Wnt4*, *Wt1*; Figure S4a) revealed that *Saa3* was specifically expressed at high levels in this cluster (Figure 1d). SAA3 is an acute‐phase protein, which, under physiological conditions, is induced in response to systemic inflammation. In the context of tumor development, SAA3 is associated with immune regulation (Hansen et al. 2015). In line with that, GO term analysis for the metastasis CAF cluster (Figure S4b) yielded the terms “Chemotaxis” (GO:0006935) and “Regulation of cytokine production” (GO:0001817).

Next, we wanted to confirm the localization of SAA3 in CAFs on tumor tissue sections using the CAF marker collagen I. Figure 1e shows the costaining of collagen I and SAA3 on lung metastasis tissue sections, clearly validating the presence of collagen I and SAA3 double‐positive CAFs. Performing the same staining on tissue sections of MM primary tumors (Figure 1e) highlighted that primary tumors were devoid of SAA3^+^ CAFs. This was consistent with the sequencing data (Figure 1d and Figure S4c) and further underlined the specificity of SAA3 in metastasis CAFs.

Since SAA3 is a secreted protein, we performed RNAscope in situ hybridization of *Saa3* RNA to prove that CAFs are the true source of *Saa3* in lung metastases. Figure 1f demonstrates that the *Saa3* probe also colocalized with collagen I and resolved the localization of *Saa3* ^+^ CAFs to be mostly at the leading edge but not within the core of the metastasis.

### 2.4 Spatial Proximity of SAA3 and NETs Suggests Crosstalk Between Metastasis CAFs and Neutrophils

**FIGURE 2:**
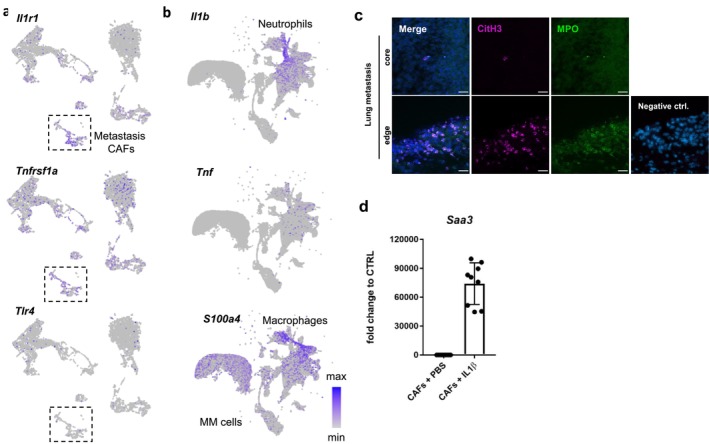
Il1b signaling as a putative activator of Saa3 expression in metastasis CAFs. (a) Expression of putatively Saa3‐inducing receptors on fibroblasts and (b) expression of corresponding ligands in all cells. The dashed rectangle in (a) highlights the metastasis CAF cluster. (c) Immunofluorescence staining of MPO and CitH3 on a lung metastasis developed in a mT/mG mouse. Time point of metastasis occurrence = 2 months after resection of the primary tumor. Scale bar = 20 μm. (d) mRNA expression of Saa3 after 6 h of IL1β treatment. Bar charts show mRNA levels as fold change to the corresponding PBS control condition of three biological replicates, respectively, with each of three technical replicates. Error bars show ± SD.

Based on our data, SAA3 may be an indicative component of a metastasis‐specific CAF population. Depending on the context, SAAs can be induced via multiple signaling pathways, among them S100A4, IL1β, and IL‐6 (Hansen et al. 2015; Djurec et al. 2018). Many of the involved receptors not only induce *Saa* expression but can also be bound by SAA protein. To gain information on the signaling pathway(s) that may induce *Saa3* expression in metastasis CAFs, we investigated the expression of putative ligand–receptor pairs in our data. Figure 2a displays exemplarily the expression of three putative receptors in the fibroblast subsets and Figure 2b the expression of the corresponding ligands in the cell‐type clusters. *S100a4* was the most prominently expressed gene within all putative ligands, however, expression of corresponding receptors was sparse, like for *Tlr4*, in metastasis CAFs. In contrast, *Tnf* and its receptor *Tnfrsf1a* were expressed in a salt‐and‐pepper pattern throughout all clusters. Interestingly, *Il1b* was strongly expressed by neutrophils, which were mainly derived from the lung metastasis samples (Figure S5), and *Il1r1* was broadly expressed on metastasis CAFs. This indicates that IL1R signaling, induced by neutrophil‐derived IL1β, might be pivotal for *Saa3* induction in metastasis CAFs.

Neutrophils have been shown to be recruited to CAF‐rich areas of human PDAC and MM and to release protumorigenic extracellular traps (t‐NETs) upon stimulation by CAF‐derived factors (Munir et al. 2021). Since SAA proteins act as chemoattractants for neutrophils (Hansen et al. 2015; Badolato et al. 1994), we costained for myeloperoxidase (MPO) and citrullinated histone H3 (CitH3) to evaluate if t‐NETs are located in the proximity to metastasis CAFs. Indeed, similar to the location of SAA3‐positive metastasis CAFs, NETs could be found close to the edge of metastasis and less frequently in the metastasis core (Figure 2c). To functionally validate that *Saa3* expression in metastasis CAFs was induced by IL1β, we isolated CAFs from murine lung metastasis and treated the cells in vitro with IL1β. While *Saa3* RNA was below the level of detection in PBS control samples, *Saa3* RNA abundance was prominent in metastasis CAFs induced by IL1β (Figure 2d). This finding was complemented by ELISA analysis of metastasis CAF cell culture supernatant, which showed that secreted SAA3 protein was enriched upon treatment with IL1β (Figure S6).

### 2.5 Human Metastasis CAFs Express SAA1 In Vivo and Upon Stimulation With IL1β In Vitro

**FIGURE 3:**
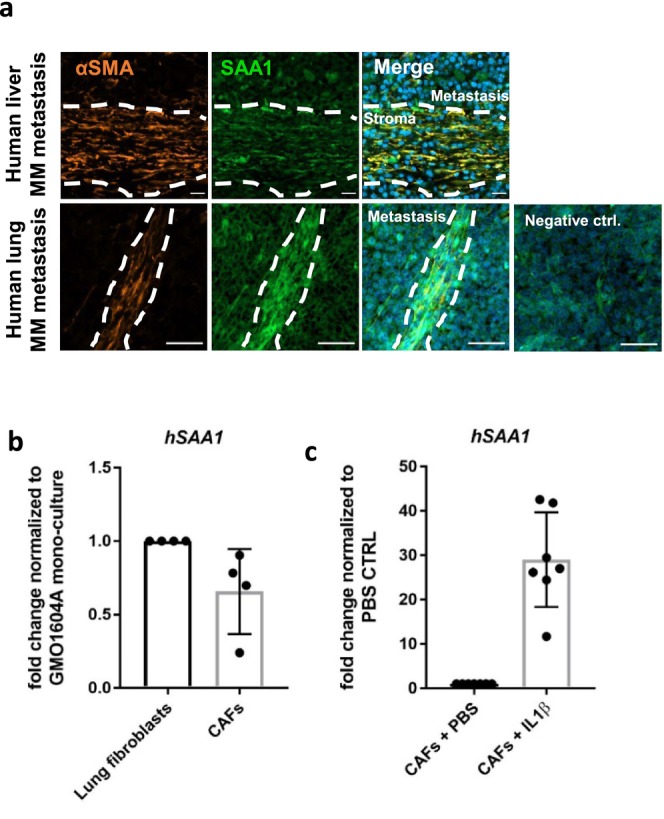
SAA1 levels in human CAFs increase upon Il1b treatment. (a) Immunofluorescence staining of aSMA and SAA1 on human lung and liver melanoma metastasis. Images represent the staining of one of three individual melanoma patient samples yielding very similar results. Scale bar = 20 μm. (b) mRNA expression of SAA1 in GMO1604A after coculture with murine lung metastasis melanoma cells. Bar charts show mRNA levels as fold change to the corresponding GMO1604A monoculture control condition of two biological replicates, respectively, with each of two technical replicates. Error bars show ± SD. (c) mRNA expression of SAA1 after 6 h of IL1β treatment. Bar charts show mRNA levels as fold change to the corresponding PBS control condition of three biological replicates, respectively, with each of two to three technical replicates. Error bars show ± SD.

The murine data suggest SAA3 as a marker of CAFs in MM metastasis and IL1β as a key element in the induction of SAA expression in these cells. To test, whether this finding can be translated into the clinical situation, we first analyzed human patient samples of melanoma metastases for the presence of SAA1, the human orthologue of SAA3 (O'Brien and Chait 2006; Kluve‐Beckerman et al. 1991). Indeed, αSMA‐positive CAFs in human lung and liver melanoma metastasis were positive for SAA1 protein (Figure 3a). Furthermore, SAA1 was enriched in CAF‐adjacent areas (Figure 3a). Based on that we wanted to know whether *SAA1* expression in human metastasis CAFs was triggered by IL1β. Therefore, we generated human metastasis CAFs in vitro and treated them with IL1β (Figure S7). Cocultivation with melanoma cells alone induced expression of the activation marker a‐SMA twofold (data not shown) but did not induce *SAA1* RNA expression in fibroblasts compared to fibroblast monoculture (Figure 3b), probably due to the presence of serum‐derived factors also known to enhance SAA1 expression masking the stimulating effect of tumor cell–derived IL1β. However, upon serum deprivation, *SAA1* levels were barely detectable, but the additional stimulus of IL1β treatment led to the upregulation of *SAA1* RNA (Figure 3c).

## 3 Discussion

Employing scRNA sequencing we have significantly expanded the current knowledge of CAF heterogeneity and have identified and characterized a previously undescribed metastasis‐specific subset of CAFs. Together with histological analyses, our data suggest crosstalk between metastasis CAFs and neutrophils based on CAF‐derived SAA3/hSAA1 and IL1β, potentially released by neutrophils, resulting in the promotion of t‐NET formation.

Established on scRNA‐sequencing analysis, CAFs are most commonly divided into immune and myofibroblastic CAFs (Ping et al. 2021). Our data unraveled transcriptional profiles of primary tumor CAFs typical for myofibroblastic CAFs and of metastasis CAFs exhibiting characteristics of immune CAFs. However, our analysis revealed more heterogeneity within these subtypes, describing the presence of so far unprecedented populations. Differences in scRNA‐sequencing platforms, varying amounts of analyzed CAFs together with a more complex tissue comparison—comparing not only one tumor site to unchallenged tissue but also to a metastatic site—might explain the differences in CAF classifications to a certain extend (Ntranos et al. 2019; Wang et al. 2021). Importantly, in addition to scRNA sequencing revealing a distinct metastasis‐specific CAF population, its presence was also confirmed at the protein level by IF staining on murine and human tissue sections. It is also important to note that in our study, as compared to previous studies, only immune cells were depleted in a subset of all replicates rendering our approach largely unbiased.

We found that MM metastasis CAFs are specifically enriched for *Saa3*. SAA3 is linked to the premetastatic niche formation (Lee and Beatty 2021). Djurec et al. (2018) showed that *Saa3*‐high CAFs have a protumorigenic function in PDAC. Friedman et al. (2020) describe a *Saa3*‐positive inflammatory CAF subset to be present also in breast cancer metastases. Here, SAA3‐positive CAFs are detectable both in early‐stage (but not late‐stage) primary tumors and metastases forming separate subgroups within the main populations. In contrast, neither early‐stage (Davidson et al. 2020) nor late‐stage (our data) MM primary tumors contain SAA3‐positive CAFs, suggesting that metastasis‐specific SAA3 expression might depend on the tumor entity.

Searching for the mode of action of metastasis CAFs, we found a putative crosstalk between neutrophils and metastasis CAFs via IL1β‐related signaling. Interestingly, IL1β was prominently expressed, while other factors, putatively released by neutrophils to recruit CAFs (e.g., TNF and TGFβ) (Sahai et al. 2020), were expressed to a much lesser extent in our model (data not shown). Neutrophils are nowadays known to be a diverse and plastic component of the TME, which can be reprogrammed toward a protumorigenic role (Xiong et al. 2021). Among the functions of neutrophils in the TME is the release of NETs. Multiple functions have been described for NETs in the context of cancer, such as enhanced trapping of circulating tumor cells in distant organs and induction of cancer cell proliferation (Xiong et al. 2021). Furthermore, Munir et al. (2021) proposed the mechanism of tumor‐induced NETosis (t‐NETosis) promoted by CAF‐derived amyloid‐beta in murine and human PDAC and MM primary tumors/metastases. We complement this idea by suggesting a second, metastasis‐specific mechanism, in which SAA3 supports the recruitment of neutrophils to the metastatic site. According to the data reported by Munir et al., NETs are less frequent in human MM primary tumors than MM liver metastases, which is in line with our hypothesis that a second, SAA1/3‐dependent, mechanism fosters t‐NETosis in MM metastasis. Thus, this regulatory circuit may represent a previously unknown option of therapeutic intervention by inhibiting IL1β signaling in metastasis CAFs via the IL1R antagonist anakinra (NCT02021422) and subsequent reduction of SAA1/3 production and abrogation of neutrophil recruitment.

Further underlining the clinical relevance of SAA proteins in the context of metastasis, SAA levels in PDAC and NSCLC patients were elevated in the presence of liver metastasis as compared to normal donors or patients with locally advanced tumors (Lee et al. 2019). Interestingly, SAA protein in the plasma of melanoma patients has been shown to be a prognostic marker for poor prognosis, already at early stages of melanoma progression (Findeisen et al. 2009). Our data indicate that increasing levels of SAA protein are not only attributable to induced expression in hepatocytes but also to SAA expression in metastasis CAFs. Moreover, this might not be linked solely to liver metastasis but also to metastases in other organs, such as the lung.

Taken together, our analysis deepens the understanding of CAFs in MM metastasis and sheds light on further putative opportunities to tackle the treatment of metastasized MM by targeting metastasis CAFs and corresponding signaling pathways.

## 4 Materials and Methods

### 4.1 MT‐Ret–Derived Model for MM

All mouse experiments were performed according to the approval of the local government authorities (Regierungspräsidium Karlsruhe, G321/19).

MT‐ret fragments (in vivo Passage 2) were expanded in vivo and biobanked as described in Gengenbacher et al. (2021). First, MT‐ret tumor fragments (Ø = 3 mm) were subcutaneously transplanted into 8‐ to 10‐week‐old mice (male and female), and tumor volume was measured by Vernier caliper at least three times a week (*V* = 0.5 × *L* × *H* × *W*; *V*: volume, *L*: length, *H*: height, and *W*: width). At a cut‐off volume of 550 mm^3^, primary tumors were surgically resected and mice were monitored for metastasis development by MRI.

mT/mG mice (Gt (ROSA)26Sor^tm4(ACTB‐tdTomato,‐EGFP)Luo/^J) (Muzumdar et al. 2007) harbor a membrane‐targeted tdTomato (mT) cassette, which is flanked by a *loxP* site on either side, leading to ubiquitous red fluorescence in all tissues and cell types. In the presence of active Cre, the mT cassette will be deleted and the membrane‐targeted EGFP (mG) cassette located downstream of the mT cassette will be expressed. This would lead to green fluorescence instead of red. Since no Cre was included in our model, all cells remained red fluorescent.

### 4.2 Histology and Immunohistochemistry

Paraffin‐embedded human melanoma tissue was provided by the Tissue Bank of the National Center for Tumor Diseases (NCT) Heidelberg, Germany.

Dissected murine tumors and metastases were fixed in 4% PFA and then (I) embedded in Tissue‐Tek O.C.T. Compound for cryosectioning; or (II) embedded in paraffin. Staining of PDPN (clone 8.1.1., Hibridoma Bank), aSMA (#F3777‐.2ML, Sigma‐Aldrich), and collagen I‐AF488 (#1310‐30, Southernbiotech; 1:100) were performed on cryosections. Staining of SAA3 (#ab233547‐1001, Abcam; 0.45 mg/mL), MPO (#AF3667, R&D; 1:50), CitH3 (#ab5103, Abcam; 1:50), and SAA1 (#MAB30191, R&D Systems, 1:50) was performed on FFPE sections after permeabilization with PBS‐Triton (0.3%) and antigen retrieval in TE buffer.

### 4.3 RNAscopeTM In Situ Hybridization and Protein Codetection

RNAscopeTM (Advanced Cell Diagnostics, ACD; Hayward, CA) was performed using a probe against murine Saa3 (RNAscope Probe—Mm‐Saa3; 7 ZZ pairs; #446841; ACD) according to manufacturer's instructions (Document Number: 323100‐USM) with integrated immunofluorescence codetection (Document Number: MK‐5150) of Type 1 collagen AF488 (#1310‐30; SouthernBiotech). The Saa3 probe was coupled to the Opal 570 fluorophore (1:1500). Fluorescence signals of the Saa3 probe and the Type 1 collagen antibody were detected using Axio Scan.Z1 (Zeiss, Oberkochen) and afterward processed with the Zen Blue Software.

### 4.4 Human IL1β In Vitro Assay

Primary human lung fibroblast cells (GMO1604A; https://www.cellosaurus.org/CVCL_7325 (Herr et al. 1994)) were cultured alone or together with primary murine lung metastasis melanoma cells (Jahangiri et al. 2019) in DMEM supplemented with 10% FCS and 1% P/S. RNA samples were collected after 4 days of coculture. For IL1β treatment, cells were starved for 12 h in DMEM supplemented with 1% P/S, followed by 6 h (RNA) or 8 h (supernatant) incubation with IL1β (#201‐LB‐010/CF, Biotechne) or PBS. The cell line was authenticated using Multiplex Cell Authentication by Multiplexion GmbH (Heidelberg, Germany). The SNP profile matched known profiles or was unique.

### 4.5 Murine IL1β In Vitro Assay

CAFs were isolated from murine lung metastasis and expanded in MEM supplemented with 15% FCS, 1% P/S, 1% NEAA, and 1% sodium pyruvate. For IL1β treatment, cells were starved for 12 h in MEM supplemented with 1% P/S, followed by 6 h (RNA) or 8 h (supernatant) incubation with IL1β (#401‐ML‐010/CF, Biotechne) or PBS.

### 4.6 RNA Isolation

Cells were harvested in QIAzol lysis reagent and stored at −80°C until RNA isolation with the miRNeasy Mini Kit (Qiagen) according to the manufacturer's instructions. Using the RNase‐Free DNase Set (Qiagen), on‐column DNA digestion was conducted following the manufacturer's manual.

### 4.7 Quantitative Real‐Time PCR

For cDNA synthesis with the RevertAid Reverse Transcriptase (200 U/μL, Thermo Fisher Scientific), up to 2 μg total RNA was used. Consecutive quantitative real‐time PCR was conducted by using the Power SYBRTM Green Master Mix (Applied Biosystems, Woolston, UK) and the StepOnePlus Real‐Time PCR system (Applied Biosystems). The following primer pairs were used: mSaa3 fw 5′‐TTGATCCTGGGAGTTGACAG‐3′; mSaa3 rev 5′‐CACTCATTGGCAAACTGGTC‐3′; hSAA1 fw 5′‐ACCATGAAGCTTCTCACGGG‐3′; rev 5′‐ATGTCCCGAGCCCCATCAAA‐3′.

### 4.8 10× Single‐Cell RNA Sequencing

Tissues of primary tumors (*n* = 5), lung metastases (*n* = 6), and unchallenged lungs (*n* = 3) were mechanically dissociated followed by incubation for 45 min, rotating in the cold room, in an enzyme mix containing LiberaseTM (#05401127007 Roche, Switzerland; 0.25 U/mL), collagenase D (#11088866001 Roche, Switzerland; 0.6 U/mL), dispase II (#17105‐041 Gibco/Thermo Fisher Scientific, USA; 4.5 U/mL), and DNaseI (Sigma Aldrich, USA; 50 U/mL). Remaining tissue fragments were further dissociated by passing the suspension through a syringe with an 18G cannula. The enzymatic reaction was stopped with DMEM containing 1% BSA and 2.5 mM EDTA. After the lysis of red blood cells, suspensions were cryopreserved in 90% FCS + 10% DMSO. The above‐described protocol was developed based on recommendations from 10× Genomics (CG00039 Rev. D) and multiple protocols described in literature (Wutschka et al. 2021; O'Flanagan et al. 2019; Guerrero‐Juarez et al. 2019; Rinkevich et al. 2015).

Prior to library preparation, cells were either sorted for living tdTomato^+^ cells or living tdTomato^+^ CD45 cells. For additional CD45^+^ cell depletion, cells were incubated with CD45^−^ APC/Cy7 antibody (30‐F11, BioLegend 103115).

Library preparation was performed using the Chromium Next GEM single‐cell 3′ GEM, Library, and Gel Bead Kit v3.1 together with the Chromium Next GEM Chip G Single Cell Kit, following the manufacturer's instructions. Sequencing of the libraries was performed by the next‐generation sequencing (NGS) Core Facility DKFZ on a NovaSeq 6000.

### 4.9 Data Processing and Quality Control

Merged FASTQ files were processed using cellranger v6.1.1 with default parameters and aligned to the mouse GRCm38 assembly. Subsequent analyses were conducted using Seurat v3.2.2 (Stuart et al. 2019).

To ensure data quality, we employed quantile‐based thresholds for individual cell assessment. Cells exceeding the upper quantile threshold (90%) for the percentage of ribosomal and mitochondrial gene counts were considered potential outliers and excluded from downstream analyses. Moreover, we evaluated the number of detected features in each cell. Cells falling below the lower quantile threshold (2.5%) or exceeding the upper quantile threshold (97.5%) were categorized as low‐quality or outlier cells, respectively, and were removed from further analyses.

For dimensionality reduction, we applied the RunUMAP function using the first 17 principal components for the entire dataset and the first 12 dimensions for the fibroblast subsetted dataset. Clustering was performed using the FindNeighbors function with the first 17 principal components and the FindClusters function with a resolution of 0.5. Marker genes for the identified clusters were determined using the FindAllMarkers function, with criteria set at > 25% of cells expressing the markers in the given cluster and a log fold change of > 0.25. To visualize gene expression levels projected onto UMAP plots, we utilized the cellcuratoR package (Voigt et al. 2020). Cluster annotation was performed using known cell‐type markers to assign biological identities to the identified cell clusters.

### 4.10 Cell Cycle Analysis

To analyze the cell cycle phase for each cell, we followed the approach described by Tirosh et al. (2016). Specifically, we assessed the relative expression of a set of 43 G1/S and 55 G2/M genes for each cell to generate a cell cycle score. Based on this score, we assigned cells to their corresponding cell cycle phase.

### 4.11 GO Term Analysis

Gene Ontology (GO) term analysis was performed by uploading the top marker genes with an adjusted *p*‐value smaller than 0.01 and an average log2 fold change bigger than 1 into the metascape online tool and analysis for GO term enrichment (Zhou et al. 2019).

## Author Contributions

**Saskia Tauch:** investigation, writing – original draft, conceptualization, writing – review and editing, funding acquisition. **Joschka Hey:** investigation, software, formal analysis, writing – review and editing. **Bettina Kast:** investigation. **Nicolas Gengenbacher:** investigation, resources. **Lena Weiß:** investigation. **Melanie Sator‐Schmitt:** investigation. **Sabrina Lohr:** investigation. **Alexander Brobeil:** resources, methodology. **Peter Schirmacher:** resources, project administration, methodology. **Jochen Utikal:** resources, funding acquisition. **Hellmut G. Augustin:** resources, methodology. **Christoph Plass:** methodology, resources, software. **Peter Angel:** conceptualization, funding acquisition, writing – original draft, writing – review and editing, project administration, supervision, resources.

## Ethics Statement

All mouse experiments were performed according to the approval of the local government authorities (Regierungspräsidium Karlsruhe, G321/19). Human material was used according to the ethical approval S‐206/2005.

## Conflicts of Interest

The authors declare no conflicts of interest.

## Supporting information


**Figure S1.** (a) Schematic overview of biobanking and procedures of the MT‐ret derived model. (b) Immunofluorescence staining of PDPN, SMA, and endogenous tdTomato on a primary tumor engrafted in a mT/mG mouse. Arrowhead indicates tdTomato‐positive CAFs. The dashed line indicates the tumor/TME margin. Time point of resection = Day 20.
**Figure S2.** (a) Proportion of different tissues within the dataset. Number of cells per tissue, which passed QC. (b) Dot plot displays the expression of selected lineage markers across all cell types. The size of the dot indicates the percentage of cells within a cluster, which express the gene. Color reflects the average expression.
**Figure S3.** (a) Expression marker genes, which were used for in silico isolation of fibroblast, across all cell types. The size of the dot indicates the percentage of cells within a cluster, which express the gene. Color reflects the average expression. (b) Representative marker genes were used to designate subclusters to fibroblast types. Color code indicates the level of gene expression. (c) Cell cycle analysis fibroblast subclusters. Color code indicates in which cell cycle phase cells were as follows: G1 (red), S (green), and G2/M (blue). Dashed line encircles highly proliferative CAF clusters. (d) Tissue of origin of fibroblast subsets. Color code indicates if a cell is derived from unchallenged lung tissues, from lung metastasis, or primary tumor tissue.
**Figure S4.** Metastasis CAF signature is marked by Saa3 expression and related signaling. (a) Violin plots show expression of the top marker genes of metastasis CAFs for all CAF subpopulations. (b) GO term analysis with the top marker genes with an adjusted *p*‐value < 0.01 and an average log2 fold change > 1 (Metascape: Zhou et al. [2019]). (c) Expression of Saa3 in all cell clusters.
**Figure S5.** TME cluster in MM and unchallenged lung tissues. (a) TME clusters extracted from Figure 1d with cells color coded for subclusters. (b) Tissue of origin of subsets. Color code indicates if a cell is derived from unchallenged lung tissues (green), lung metastasis (blue), or primary tumor tissue (purple).
**Figure S6.** SAA3 protein concentration in murine CAFs increases upon Il1β treatment protein concentration of SAA3 after 8 h of IL1β treatment and the corresponding PBS control condition of three biological replicates, respectively. Error bars show ± SD.
**Figure S7.** Schematics of coculture and IL1β treatment.

**Table S1.** Marker genes for MM and TME cell clusters. This table lists the marker genes, which were employed for the identification of MM and TME cell clusters.

**Table S2.** Differentially expressed genes of the described fibroblast and CAF clusters. This table lists the differentially expressed genes for the identified fibroblast and CAF clusters.

Data S1.

## Data Availability

Raw single‐cell RNA‐seq data and corresponding UMI count matrices are deposited in GEO, Accession Number GSE254072 (https://www.ncbi.nlm.nih.gov/geo/query/acc.cgi?acc=GSE254072).
