# Supplementary figures and images for: A Unique Signature for Cancer‐Associated Fibroblasts in Melanoma Metastases
Source: Pigment Cell Melanoma Res. 2025 Feb 9;38(2):e70002. doi: 10.1111/pcmr.70002 (PMC11808227; doi:10.1111/pcmr.70002)

## Slide 1
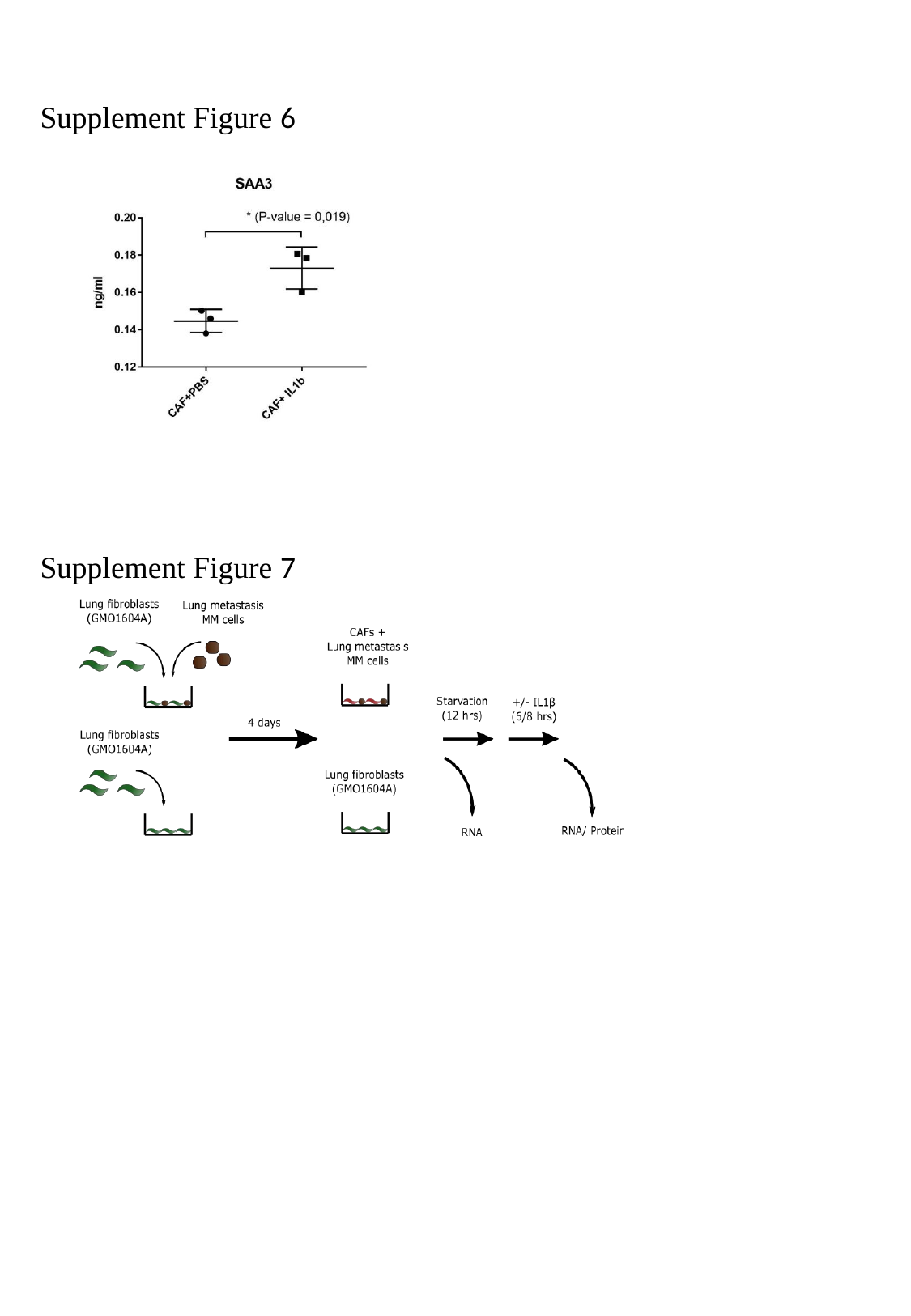

Supplement Figure 6
Supplement Figure 7

Supplement: Supplementary file 1 — Figure S1. (a) Schematic overview of biobanking and procedures of the MT‐ret derived model. (b) Immunofluorescence staining of PDPN, SMA, and endogenous tdTomato on a primary tumor engrafted in a mT/mG mouse. Arrowhead indicates tdTomato‐positive CAFs. The dashed line indicates the tumor/TME margin. Time point of resection = Day 20. Figure S2. (a) Proportion of different tissues within the dataset. Number of cells per tissue, which passed QC. (b) Dot plot displays the expression of selected lineage markers across all cell types. The size of the dot indicates the percentage of cells within a cluster, which express the gene. Color reflects the average expression. Figure S3. (a) Expression marker genes, which were used for in silico isolation of fibroblast, across all cell types. The size of the dot indicates the percentage of cells within a cluster, which express the gene. Color reflects the average expression. (b) Representative marker genes were used to designate subclusters to fibroblast types. Color code indicates the level of gene expression. (c) Cell cycle analysis fibroblast subclusters. Color code indicates in which cell cycle phase cells were as follows: G1 (red), S (green), and G2/M (blue). Dashed line encircles highly proliferative CAF clusters. (d) Tissue of origin of fibroblast subsets. Color code indicates if a cell is derived from unchallenged lung tissues, from lung metastasis, or primary tumor tissue. Figure S4. Metastasis CAF signature is marked by Saa3 expression and related signaling. (a) Violin plots show expression of the top marker genes of metastasis CAFs for all CAF subpopulations. (b) GO term analysis with the top marker genes with an adjusted p‐value < 0.01 and an average log2 fold change > 1 (Metascape: Zhou et al. [2019]). (c) Expression of Saa3 in all cell clusters. Figure S5. TME cluster in MM and unchallenged lung tissues. (a) TME clusters extracted from Figure 1d with cells color coded for subclusters. (b) Tissue of origin of subsets. [file PCMR-38-0-s003.zip › Supplement_Figures_6-7_Tauch_et_al_PCMR_revised.pptx]
