# Supplementary material for: A Unique Signature for Cancer‐Associated Fibroblasts in Melanoma Metastases
Source: Pigment Cell Melanoma Res. 2025 Feb 9;38(2):e70002. doi: 10.1111/pcmr.70002 (PMC11808227; doi:10.1111/pcmr.70002)
Supplement: Supplementary file 4 — Data S1. [file PCMR-38-0-s001.docx]

**SUPPLEMENTARY MATERIAL**

**ELISA**

The supernatant was harvested, centrifuged for 20 min at 1000 x g and stored at -80°C. The ELISA was performed with the Sandwich ELISA Kit for Serum Amyloid A3 (#ABIN6973552, antibodies-online) according to the manufacturer's instructions.
